# Supplementary material for: A Case Report of Secondary Syphilis Co-Infected with Measles: A Diagnostic Dilemma with Fever and Rash
Source: Trop Med Infect Dis. 2022 May 9;7(5):70. doi: 10.3390/tropicalmed7050070 (PMC9144679; doi:10.3390/tropicalmed7050070)
Supplement: Supplementary file 1 [file tropicalmed-07-00070-s001.zip › tropicalmed-1691427-supplementary.pdf]

Tropical Medicine and Infectious Disease

Case Report

A Case Report of Secondary Syphilis Co-infected with Measles: a Diagnostic Dilemma with Fever and Rash.

Hisham Ahmed Imad [1,2,3,*], Ploi Lakanavisid [2,4], Phimphan Pisutsan [2,5], Kentaro Trerattanavong [6], Thundon Ngamprasertchai [2,5], Wasin Matsee [2,5], Watcharapong Piyaphanee [2,5], Pornsawan Leaungwutiwong [7,8], Wang Nguitragool [1,9], Emi E. Nakayama [3] and Tatsuo Shioda [3]

1 Mahidol Vivax Research Unit, Faculty of Tropical Medicine, Mahidol University, Bangkok 10400, Thailand; wang.ngu@mahidol.edu (W.N.)
2 Thai Travel Clinic, Hospital for Tropical Diseases, Faculty of Tropical Medicine, Mahidol University, Bangkok 10400, Thailand; ploi.la@go.buu.ac.th (P.L.); phimphan@thaitravelclinic.com (P.P.); thundon.ngm@mahidol.ac.th (T.N.); wasin.mat@mahidol.edu (W.M.); watcharapong.piy@mahidol.ac.th (W.P.)
3 Center for Infectious Disease Education and Research, Department of Viral Infections, Research Institute for Microbial Diseases, Osaka University, Suita 565-0871, Osaka, Japan; emien@biken.osaka-u.ac.jp (E.E.N.); shioda@biken.osaka-u.ac.jp (T.S.)
4 Department of Preventive Medicine and Family Medicine, Faculty of Medicine, Burapha University, Chonburi 20130, Thailand
5 Department of Clinical Tropical Medicine, Faculty of Tropical Medicine, Mahidol University, Bangkok 10400, Thailand
6 Hull York Medical School, University road, Heslington, York, YO10 5DD, UK; hykt2@hyms.ac.uk (K.T.)
7 Tropical Medicine Diagnostic Reference Laboratory, Faculty of Tropical Medicine, Mahidol University, Bangkok 10400, Thailand; pornsawan.lea@mahidol.ac.th (P.L.)
8 Department of Microbiology and Immunology, Faculty of Tropical Medicine, Mahidol University, Bangkok 10400, Thailand
9 Department of Molecular Tropical Medicine and Genetics, Faculty of Tropical Medicine, Mahidol University, Bangkok 10400, Thailand
* Correspondence: hishamahmed.ima@mahidol.ac; Tel.: +66-631501402

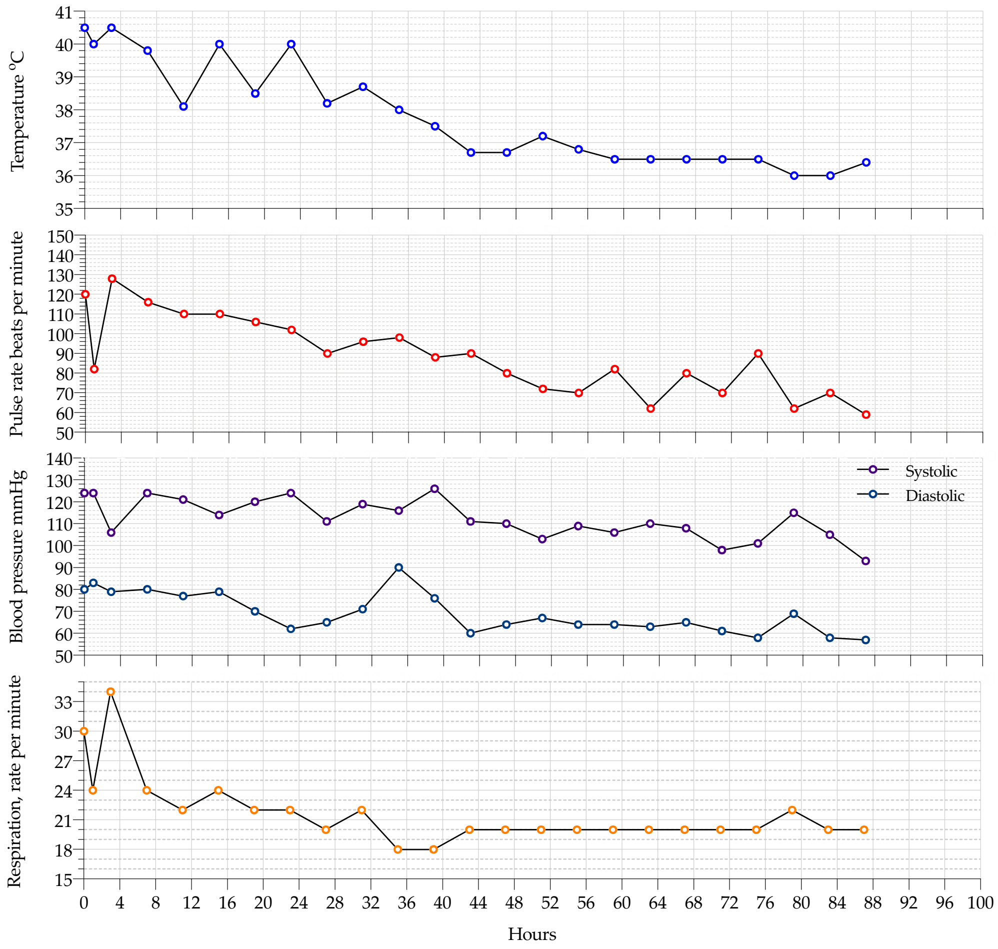

Figure S1. The kinetics of vital signs parameters demonstrating the clinical course of the illness.
